# Performance of an artificial intelligence algorithm for interpreting lung sounds from children hospitalised with pneumonia in Malawi

**DOI:** 10.7189/jogh.15.04264

**Published:** 2025-09-19

**Authors:** Nadia E Hoekstra, Maganizo B Chagomerana, Zachary H Smith, Annapurna Kala, Ian McLane, Charl Verwey, Daniel Olson, W Chris Buck, Justin Mulindwa, Alex Gaudio, Sunaina Kapoor, Holly B Schuh, Msandeni Chiume, Elizabeth Fitzgerald, Mounya Elhilali, Tisungane Mvalo, Mina Hosseinipour, Eric D McCollum

**Affiliations:** 1 Department of Pediatrics, University of North Carolina School of Medicine, Chapel Hill, North Carolina, USA; 2 Global Program in Pediatric Respiratory Sciences, Eudowood Division of Pediatric Respiratory Sciences, Johns Hopkins University School of Medicine, Baltimore, Maryland, USA; 3 University of North Carolina Project Malawi, Lilongwe, Malawi; 4 Department of Medicine, University of North Carolina School of Medicine, Chapel Hill, North Carolina, USA; 5 Department of Pediatrics, Division of Pediatric Critical Care Medicine, Kaiser Permanente Northern California, Oakland, California, USA; 6 Department of Electrical and Computer Engineering, Johns Hopkins University, Baltimore, Maryland, USA; 7 Department of Paediatrics and Child Health, Faculty of Health Sciences, University of the Witwatersrand, Johannesburg, South Africa; 8 Department of Pediatrics, University of Colorado School of Medicine, Aurora, Colorado, USA; 9 University of California Los Angeles, David Geffen School of Medicine, Los Angeles, California, USA; 10 University Teaching Hospital, Lusaka, Zambia; 11 Department of Epidemiology, Johns Hopkins Bloomberg School of Public Health, Baltimore, Maryland, USA; 12 Department of Paediatrics, Kamuzu Central Hospital, Lilongwe, Malawi; 13 Department of International Health, Johns Hopkins Bloomberg School of Public Health, Baltimore, Maryland, USA

## Abstract

**Background:**

Pneumonia is a leading cause of death in under five year olds globally. World Health Organization (WHO) pneumonia diagnostic guidelines rely on non-specific clinical findings. Lung auscultation could improve pneumonia diagnosis, but conventional stethoscopes have implementation challenges. To address this, we developed an artificial intelligence (AI)-enabled digital auscultation system. We evaluated the system’s AI lung sound analysis algorithm in children with severe pneumonia in Malawi.

**Methods:**

We enrolled children aged 2–59 months hospitalised with WHO-defined severe pneumonia. A study physician recorded lung sounds with a digital stethoscope at six chest positions. Recordings were de-identified, filtered, and interpreted by a trained and certified physician listening panel. Interpretable recordings were analysed by the AI algorithm. We evaluated the agreement of normal (absence of adventitial lung sounds) *vs*. abnormal (presence of adventitial lung sounds) classifications, by chest position and by patient, between the AI algorithm and the listening panel using raw percent agreement kappa statistics, both unadjusted and adjusted for chance agreement.

**Results:**

We enrolled 100 children and analysed 95 with interpretable recordings. The median age was 12.6 months (interquartile range (IQR) = 5.4, 19.0) and 54% (51 / 95) were female. Among interpretable recordings, 59.2% (294 / 497) of chest positions were abnormal per the listening panel compared to 52.7% (262 / 497) per the AI algorithm. The listening panel and AI algorithm agreed on classifications in 83.1% (413 / 497) of chest positions (unadjusted kappa 0.7; adjusted kappa 0.7) and 91.6% (87/95) of patients (unadjusted kappa 0.7; adjusted kappa 0.8). The AI algorithm’s sensitivity and specificity for identifying abnormal lung sounds, compared to the listening panel, were 80.3% and 87.2% for chest positions and 96.3%, and 66.7% for patients.

**Conclusions:**

This AI lung sound classification algorithm accurately identified abnormal lung sounds in children with severe pneumonia. Next steps include training the algorithm to identify uninterpretable recordings and different abnormal sounds.

Pneumonia remains the leading infectious cause of mortality in children under five worldwide [1,2]. The World Health Organization (WHO) Integrated Management of Childhood Illnesses (IMCI) guidelines provide the current standard for managing paediatric pneumonia in low-income and middle-income countries (LMICs) [3]. Developed in the 1980s, the IMCI algorithm enables non-physician health care workers (HCWs) to identify children with likely bacterial pneumonia [3]. By increasing antibiotic treatment, IMCI has reduced paediatric pneumonia deaths over the past two decades [3,4]. In 2013, the IMCI approach was broadened to include hospitalised children and to strengthen diagnosis and treatment protocols [5]. Despite its success, recent studies have raised concerns about low diagnostic specificity due to reliance on clinical findings, such as chest wall retractions and elevated respiratory rates, which are seen in diseases beyond bacterial pneumonia [6–9]. Overdiagnosis exposes children to antibiotics, interventions, and avoidable costs, and delays treatment. This issue is critical given rising antimicrobial resistance and a shift towards viral respiratory diseases, largely due to effective vaccines against *Haemophilus influenzae* type B and *Streptococcus pneumoniae* [10–12]. These concerns highlight the need to develop new diagnostic technologies to improve IMCI’s diagnostic performance.

Stethoscopes are a low-cost, non-invasive tool used in resource-rich settings to listen to and interpret lung sounds, and they have the potential to enhance IMCI’s diagnostic accuracy. However, lung auscultation in children by HCWs remains subjective, with only moderate levels of agreement between examiners [13,14]. Lung auscultation in children is particularly challenging due to inconsistent patient cooperation, upper airway sound transmission, variable tidal volumes, and short respiratory cycles. Successful lung auscultation is challenging in clinical settings in LMICs where most paediatric patients are managed by non-physician HCWs [15]. In LMICs, there are limited numbers of HCWs who can effectively teach and perform this skill, and background noise in high-volume areas hinders auscultation.

Emerging technologies like digital stethoscopes and artificial intelligence (AI)-enabled lung sound analysis may overcome these barriers in paediatric auscultation. Digital stethoscopes, which transmit, filter, and amplify sounds, may help HCWs in LMICs better auscultate and evaluate lung sounds in children [16]. More importantly, AI lung sound analysis can classify lung sounds for HCWs with limited training, providing an automated solution to conventional lung auscultation challenges. Adventitious lung sounds, such as crackles and wheezes, are abnormal breath sounds that may indicate underlying respiratory pathology. These sounds have unique acoustic characteristics that can be identified by AI applications [17,18]. These tools could improve diagnostic accuracy and reduce the subjectivity associated with conventional stethoscopes. Although literature exists regarding AI classification of lung sounds, few studies focus on children in LMICs, where the pneumonia burden is highest [2].

To address this gap, in 2012 we embarked on a multi-disciplinary collaboration, involving paediatricians, paediatric pulmonologists, and sound engineers to develop a digital auscultation system designed for use in children in LMICs. The system consists of three elements: a prototype digital stethoscope, automated ambient noise filtering software, and an AI lung sound analysis algorithm. While we have previously reported on this system at various developmental stages, we have yet to evaluate the AI lung sound analysis algorithm’s performance on prospectively collected data from children in real-world LMIC contexts. Therefore, we aimed to evaluate the performance of the system’s AI lung sound analysis algorithm, compared to a reference physician listening panel, in classifying lung sound recordings from hospitalised Malawian children aged 2–59 months with WHO-defined severe pneumonia.

## METHODS

### Study design and setting

This cross-sectional study was conducted at Kamuzu Central Hospital (KCH) in Lilongwe, Malawi. KCH is a government tertiary referral hospital for the Central Region of Malawi.

### Enrollment and participant eligibility

Children aged two to 59 months admitted to the paediatric ward of KCH and diagnosed with severe pneumonia were eligible. A paediatrician and a nurse reviewed all patients admitted to the ward daily to determine study eligibility. Eligible children were hospitalised for less than 24 hours and met the WHO severe pneumonia definition. Severe pneumonia was defined as the presence of cough or difficulty breathing plus any of the following: oxygen saturation <90%, central cyanosis, severe respiratory distress with grunting or severe chest indrawing, or a general danger sign (inability to feed, lethargy, reduced level of consciousness, or convulsions) [4]. Exclusion criteria included wheezing that improved after bronchodilators, chronic lung disease other than asthma or reactive airways, medical instability, tracheostomy, or invasive or non-invasive ventilation.

### Demographic, clinical and laboratory evaluation

At enrolment, demographic information, medical history, clinical data, and physical examination findings were obtained. All children received a malaria Rapid Diagnostic Test (RDT) and HIV counselling and testing as recommended by the Malawi Ministry of Health guidelines. Malaria RDT and HIV results (infected, uninfected, exposed, or unknown) were documented.

### Lung sound auscultation and recording

An American Board of Pediatrics-eligible physician used a validated methodology to record lung sounds at six sequential anatomic chest positions, for at least 10 seconds per position (Figure S1 in the **Online Supplementary Document**), using a prototype digital stethoscope [19] connected to a Zoom H4n Pro Portable Recorder®. Each participant’s recording, containing all six chest position recordings, was deidentified, uploaded to a secure computer server at the study site, and segmented by chest position using Audacity® audio editing software. Each chest position recording captured a minimum of three respiratory cycles. Participant factors during auscultation, such as motion and phonation, were documented by the study clinician. Following deidentification and segmentation, the recordings were securely transferred to Johns Hopkins University where ambient noise filtering was applied. This noise-cancellation software utilises digital stethoscope microphones to filter ambient signals from pulmonary sounds [20-22].

### Chest position recording classification by a physician listening panel

**Figure 1:**
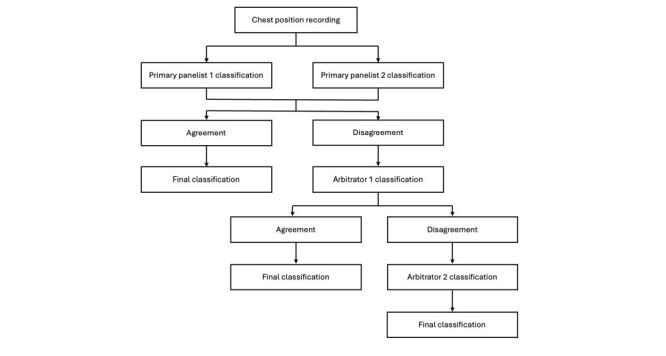
Chest position lung sound recording interpretation schema by the physician listening panel.

De-identified, segmented, and denoised chest position recordings were randomised and distributed to a trained physician listening panel. The panel consisted of three primary panelists and two arbitrators, all trained to interpret lung sounds using our validated methodology (**Figure 1**) [23]. All panelists were physicians with advanced training in either Paediatrics or Paediatric Pulmonology and had passed a certification test prior to interpreting recorded lung sounds. Each recording was randomly assigned to two primary panelists for lung sound classification. Panelists reviewed lung sounds using Audacity software, classifying recordings as interpretable or uninterpretable. Interpretable recordings were then classified as normal or abnormal, with abnormal recordings receiving an additional classification of wheezes, crackles, or both wheezes and crackles. Classification agreement between the two primary panelists resulted in a final classification label. Panelists were masked to participant clinical data and other panelists’ classifications. Recordings where primary panelists disagreed were interpreted by an initial arbitrator, who was also masked to all clinical data and other panelists’ classifications. If the arbitrator agreed with one of the primary panelists, the classification was finalised. A second arbitrator, a pediatric pulmonologist with extensive experience in digital lung auscultation, reviewed recordings where disagreement persisted between the initial arbitrator and both primary panelists. The secondary arbitrator’s classification served as the final classification, even if it disagreed with the other panelists.

### Chest position recording classification by the AI algorithm

The AI algorithm generated a classification for each denoised, interpretable chest position recording. This algorithm consists of two main components: a convolutional neural network (CNN) encoder, followed by a recurrent classifier. The CNN encoder applies multiple successive layers of filters at varying scales. Each layer captures local and increasingly abstract spectral and temporal acoustic features while reducing irrelevant noise or artifacts. The resultant condensed representation of the lung sound is passed to the recurrent classifier that examines the temporal dynamics of these extracted features, effectively capturing how the identified patterns evolve over time. The model’s robustness was ensured through cross-validation, where the model was trained on various subsets of physician listening panel annotations. During this process, the data were divided into multiple testing sets to ensure robust evaluation of the model’s performance. The algorithm was designed to classify interpretable lung sounds with at least one audible breath cycle as normal or abnormal. As training annotations were available for interpretable sounds only, the algorithm has not been trained or tested on uninterpretable samples. We combined the classifications from five algorithm iterations, using the majority output to make a final decision about each recording.

### Patient lung sound classification

Chest positions classified as interpretable by the physician listening panel were regrouped to generate patient-level classifications of normal or abnormal. Specifically, for a given patient, if all chest positions were classified as uninterpretable, then the patient classification was uninterpretable. Alternatively, if all chest positions were classified as normal, the patient classification was normal. If a patient had one or more abnormal chest positions, then the patient classification was abnormal.

### Conventional lung sound auscultation

The study clinician performed conventional lung auscultation using a Littman Classic II Infant Stethoscope® at the same six chest positions immediately prior to the recording procedure. Following the same lung sound interpretation methodology, chest positions were classified in real-time by the clinician as interpretable or uninterpretable, with interpretable lung sounds further classified as normal or abnormal. Abnormal sounds included wheezes, crackles, or wheezes and crackles.

### Statistical analysis

The sample size was determined based on the minimum acceptable agreement between the physician listening panel and the AI algorithm. Assuming an expected agreement rate of 70%, an intra-class correlation coefficient of 0.7, and a cluster size of 6 recordings per participant, a total of 91 participants was calculated to be sufficient to achieve a precision of 20% with 95% confidence. To accommodate potential data loss due to uninterpretable recordings or technical issues, the sample size was increased to 100 participants.

The analytic data set included all children with interpretable lung sound recordings, as classified by the physician listening panel. Descriptive statistics were used to summarise demographic information, medical history, and clinical data. Variables with a normal distribution were described using the mean and standard deviation (SD), while non-normally distributed variables were summarised using the median and interquartile range (IQR).

As a sensitivity analysis, we compared the demographic information, medical history, and clinical data of participants with no interpretable lung sound recordings to those with interpretable lung sound recordings. We used the χ^2^ test to compare categorical variables and the Wilcoxon rank sum test for continuous variables.

The primary outcome was pairwise agreement of normal *vs*. abnormal chest position classifications between the AI algorithm and the physician listening panel. The secondary outcome was pairwise agreement of normal and abnormal patient classifications between the AI algorithm and physician listening panel. As a sensitivity analysis, we evaluated pair-wise agreement of patient classifications between the study clinician’s conventional stethoscope interpretations and the physician listening panel, and between the study clinician and the AI algorithm.

Agreement was measured by raw percentage, Cohen’s kappa statistic, and the Brennan and Prediger statistic, which adjusts for chance agreement [24]. The strength of agreement was defined as poor (≤0), slight (0.01, 0.19) fair (0.20, 0.39), moderate (0.40, 0.59), substantial (0.60, 0.79), or almost perfect (0.80, 1.0) [23,25]. We assessed the AI algorithm's performance in detecting abnormal lung sounds, considering the physician listening panel as the reference standard, for both chest position and patient classifications, by calculating sensitivity, specificity, positive and negative predictive values, positive and negative likelihood ratios, and diagnostic odds ratio (OR). The diagnostic OR measures a test’s diagnostic performance by calculating the ratio of the odds of a positive test among diseased participants to the odds of a positive test among healthy participants [26]. We similarly assessed the AI algorithm’s performance when considering the clinician as the reference standard.

We used generalised estimating equations with an exchangeable correlation matrix and binomial distribution with logit link function models to evaluate associations between participant characteristics and raw percentage agreement *vs*. disagreement in chest position classifications between the AI algorithm and the physician listening panel. Logistic regression was used to evaluate associations between participant characteristics and patient classification agreement between the AI algorithm and the physician listening panel.

## RESULTS

**Figure 2:**
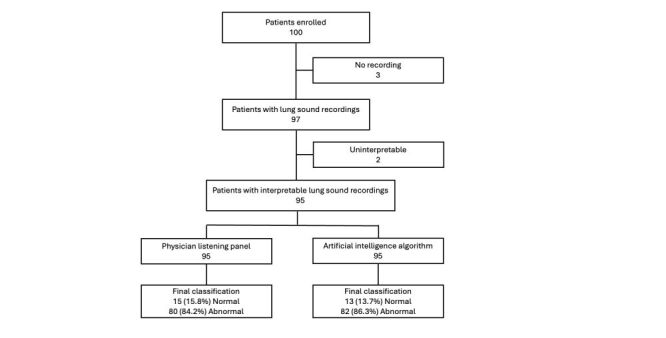
Artificial intelligence and physician listening panel lung sound classifications by patient.

**Figure 3:**
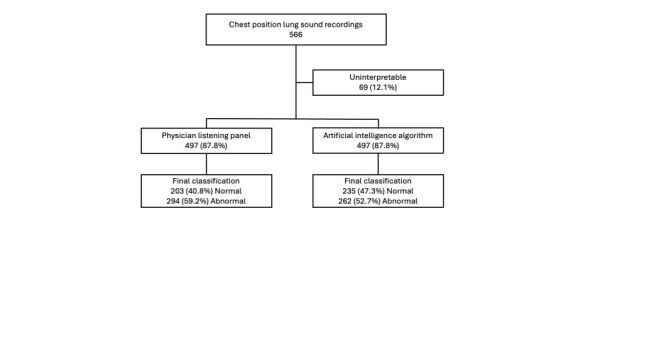
Artificial intelligence and physician listening panel lung sound classifications by chest position. Note: 4/570 (0.7%) recordings from the 95 participants with interpretable lung sound recordings were missing.

Of the 100 patients enrolled, 95 (95%) had lung sound recordings available and interpretable (**Figure 2**). Of 566 chest position recordings, 497 (88%) were interpretable (**Figure 3**). The median age of analysed participants was 12.6 months (IQR = 5.4, 19.0), with seven (7%) born prematurely. Seven (7%) patients were HIV-exposed, and 11 (12%) tested malaria positive (**Table 1**). The average WHO weight-for-height Z-score was −0.3 with an SD of 1.2. Sensitivity analysis comparing participants with no interpretable recordings to those with interpretable recordings showed no differences in demographic information, medical history, or clinical data (Table S1 in the **Online Supplementary Document**).

**Table 1:** Characteristics of children with World Health Organization-defined severe pneumonia

Characteristics	Participants (n = 95)
**Demographics**	
Age in months, median (IQR)	12.6 (5.4, 19.0)
Age categories in months, n (%)	
*2–11*	46 (48)
*12–23*	32 (34)
*24–59*	17 (18)
Females, n (%)	51 (54)
Past medical history	
Prematurity*, n (%)	7 (7)
*Tuberculosis contacts, n (%)*	6 (6)
*History of tuberculosis, n (%)*	1 (1)
*Vaccinations up to date†, n (%)*	86 (90)
**Hospitalisation characteristics**	
HIV status, n (%)	
*Infected*	0 (0)
*Uninfected*	77 (81)
*Exposed*	7 (7)
*Unknown*	11 (12)
Malaria positive‡, n (%)	11 (12)§
Corticosteroid treatment, n (%)	8 (8)
Bronchodilator treatment	21 (22)
**Clinical features**	
WHO Weight-for-height Z score, mean (SD)	−0.3 (1.2)
Axillary temperature in degrees Celsius, median (IQR)	36.8 (36.4, 37.5)
Respiratory rate in breaths/min, mean (SD)	54.4 (14.7)
SpO_2_ in room air, median (IQR)	95.0 (92.0, 97.0)

IQR – indicates interquartile range, SD – standard deviation, SpO_2_ – peripheral arterial oxyhemoglobin saturation, WHO – World Health Organization,

*By mother’s verbal report.

†Up-to-date if documented in the child’s health passport or verbally confirmed by guardian.

‡Positive rapid diagnostic test.

§27 (28%) with missing data.

Of 497 interpretable chest position recordings, 294 (59.2%) were classified as abnormal by the physician listening panel compared to 262 (52.7%) by the AI algorithm (**Figure 2**). The physician listening panel and the AI algorithm agreed on 83.1% (413 / 497) of chest position classifications (**Table 2**). The agreement beyond what is expected by chance, measured by both Cohen’s kappa and adjusted kappa statistics, was substantial for interpretable chest position recordings with or without abnormal lung sounds (**Table 2**).

**Table 2:** Lung sound classification agreement on abnormal *vs*. normal lung sounds between the artificial intelligence algorithm and physician listening panel for chest positions and patients

Normal or abnormal	Chest position classification, n = 497	Patient classification, n = 95
	
Agreement, n (%)	413 (83.1)	87 (91.6)
Kappa Statistic (95% CI)	0.659 (0.592, 0.725)	0.665 (0.447, 0.884)
Adjusted Kappa Statistic* (95% CI)	0.662 (0.596, 0.728)	0.832 (0.718, 0.945)

CI – confidence interval

*Brennan and Prediger statistic.

At the patient level, 80 / 95 patients (84.2%) had an abnormal classification by the listening panel compared to 82 / 95 (86.3%) classified by the AI algorithm (**Figure 3**), resulting in an agreement of 91.6% (87/95) (**Table 2**). Agreement by the adjusted kappa statistic was almost perfect for interpretable patient classifications with or without abnormal lung sounds (**Table 2**).

The AI algorithm demonstrated a sensitivity of 80.3% (95% CI = 75.3, 84.7) and a specificity of 87.2% (95% CI = 81.8, 91.5) in detecting abnormal lung sounds in chest position recordings, compared to the physician listening panel. The diagnostic OR of the AI algorithm indicates it was 27.7 times more likely to classify chest position recordings as abnormal and 52.3 times more likely to classify patients as abnormal when the physician listening panel also classified them as abnormal, compared to when they classified them as normal (**Table 3**).

**Table 3:** Performance of artificial intelligence algorithm for detecting abnormal lung sounds in children with World Health Organization-defined severe pneumonia in Malawi using the physician listening panel as the reference

	AI algorithm performance							
	**Abnormal classification, n/N (%)**	**Sensitivity, % (95% CI)**	**Specificity, % (95% CI)**	**PPV, % (95% CI)**	**NPV, % (95% CI)**	**LR+ (95% CI)**	**LR- (95% CI)**	**Diagnostic OR (95% CI)**
Chest position classification	262/497 (52.7)	80.3 (75.3, 84.7)	87.2 (81.8, 91.5)	90.1 (85.8–93.4)	75.3 (69.3, 80.7)	6.27 (4.36, 9.01)	0.23 (0.18, 0.29)	27.7 (16.8, 45.7)
Patient classification	82/95 (86.3)	96.3 (89.4, 99.2)	66.7 (38.4– 88.2)	93.9 (86.3, 98.0)	76.9 (46.2, 65.0)	2.89 (1.41, 5.91)	0.06 (0.02, 0.18)	51.3 (11.2, 234)

AI – artificial intelligence, CI – confidence interval, diagnostic OR – diagnostic odds ratio, LR+ – positive likelihood ratio, LR- – negative likelihood ratio, NPV – negative predictive value, PPV – positive predictive value

Predictors of raw percentage agreement between the AI algorithm and the physician listening panel for chest position classifications and patient-level classifications are presented in Table S3–4 in the **Online Supplementary Document**. Children who were uncooperative during lung auscultation had 49% lower odds of agreement for chest position classifications compared to cooperative children (Table S3 in the **Online Supplementary Document**). No factors were significantly associated with agreement for patient-level classifications (Table S4 in the **Online Supplementary Document**).

## DISCUSSION

We evaluated a novel digital auscultation system, which includes a prototype digital stethoscope, filters for ambient noise removal, and an AI algorithm developed to analyse lung sounds in acutely ill children in LMIC clinical contexts with dynamic noise environments. Our data demonstrate that the AI algorithm can accurately identify abnormal lung sounds in children with severe pneumonia in a paediatric ward in Malawi, showing high discriminatory power and substantial reliability compared to a physician listening panel’s reference standard interpretations of the same recordings. These results represent an important step forward for the potential application of AI-powered digital auscultation systems in identifying abnormal lung sounds in acutely ill children in real-world clinical contexts where HCWs lack training to effectively perform lung auscultation.

The performance of our AI algorithm compares favourably with existing paediatric literature on automated lung sound analysis. A 2022 systematic review of 10 studies across multiple countries evaluated the effectiveness of digital auscultation with automated lung sound analysis compared to conventional physician lung auscultation for pneumonia diagnosis in ~ 3000 children [27]. The review found a wide range of accuracies for classifying adventitious (abnormal) lung sounds, from 66.3 to 100% [27]. Children in the reviewed studies had various clinical conditions, including pneumonia and other respiratory conditions. Our AI algorithm achieved an accuracy of 83.1% for chest positions and 91.6% for patients in classifying abnormal lung sounds in children with severe pneumonia in a challenging environment with high ambient noise levels. To our knowledge, few studies have shown that an AI algorithm can achieve high accuracy in classifying lung sounds in children with pulmonary disease in this type of real-world context [28], further supporting the potential usability for our diagnostic platform.

In its current version, the AI algorithm of our digital auscultation system has several limitations. First, the algorithm cannot yet classify a recording as uninterpretable, as it maps the signal onto one of two possible cases: normal or abnormal. Consequently, the HCW using this system must judge the quality of the measurement in real time. The algorithm requires further development to instruct the user to repeat the measurement when there are no audible breath sounds, which may occur due to background noise or movement artifacts, both common during chest auscultation in sick, agitated children. Second, the AI results are not generated in real-time, which is necessary for clinical application. Third, the algorithm cannot yet differentiate between adventitious (abnormal) sounds, such as high or low-pitched wheezes and fine or coarse crackles, which may represent different underlying disease processes and require different treatment. Fourth, the algorithm is also unable to distinguish upper respiratory sounds (like stridor, stertor, or vocalisations) from lower respiratory sounds, potentially leading to misclassification of recordings. Lastly, the algorithm may perform less accurately when exposed to new patient data sets. To overcome this limitation, it may be necessary to recalibrate the algorithm when introduced into new contexts, including other LMIC settings that have different patient demographics or ambient noise profiles. Feasible implementation strategies, such as algorithm ‘fine-tuning’ or retraining using new data, could be utilised.

The clinical use-case of this digital auscultation system deserves discussion, as its application in the management of children with non-severe pneumonia is clearer than in severe pneumonia. For patients with non-severe pneumonia, a ‘normal *vs*. abnormal’ AI algorithm may help determine which children require immediate antibiotic treatment and which can be safely observed without antibiotics. This could help save valuable resources and promote antibiotic stewardship in settings with resource limitations. Indeed, the randomised, double-blinded placebo trial BLAAAST (Bangladesh Lung Auscultation with Artificial Intelligence for Antibiotic Stewardship) seeks to address this important question for children aged 2–59 months in Bangladesh. However, in hospitalised patients with WHO-defined severe pneumonia, where the risk of poor outcomes is higher and clinicians are more likely to treat empirically with antibiotics, a binary classification may have limited impact on clinical decision-making. In such scenarios, an AI algorithm capable or further differentiating between adventitious sounds such as wheezes and crackles may be more beneficial for clinical management. For instance, crackles may be associated with bacterial pathogens that require antibiotics, whereas wheezing may indicate viral-induced airway inflammation that does not need antibiotics. We previously reported that crackles identified by a human listening panel are associated with higher odds of radiographic pneumonia and mortality, while wheezes are associated with lower odds of radiographic pneumonia and mortality, consistent with this clinical framework [29–31]. We acknowledge that there can be clinical overlap between wheezes and crackles, as some patients with wheezing may also have a secondary bacterial infection.

## CONCLUSIONS

In summary, this novel digital auscultation system, which includes a prototype digital stethoscope, ambient noise filtering, and an AI lung sound classification algorithm, shows potential for accurately identifying abnormal lung sounds in children with pneumonia. The next steps include further developing the algorithm to classify lung sounds as uninterpretable and to distinguish between wheezes, crackles, and upper respiratory sounds. Additionally, it will be important to evaluate how the AI algorithm’s lung sound findings relate to the outcomes of children with pneumonia in LMICs.

## Additional material


Online Supplementary Document
