# Supplementary material for: Performance of an artificial intelligence algorithm for interpreting lung sounds from children hospitalised with pneumonia in Malawi
Source: J Glob Health. 2025 Sep 19;15:04264. doi: 10.7189/jogh.15.04264 (PMC12447016; doi:10.7189/jogh.15.04264)
Supplement: Online Supplementary Document [file jogh-15-04264-s001.pdf]

Supplement to: Hoekstra NE, Chagomerana MB, Smith ZH, Kala A, McLane I, Verwey C, Olson D, Buck WC, Mulindwa J, Gaudio A, Kapoor S, Schuh HB, Chiume M, Fitzgerald E, Elhilali M, Mvalo T, Hosseinipour M, McCollum ED. Performance of an artificial intelligence algorithm for interpreting lung sounds from children hospitalized with pneumonia in Malawi. J Glob Health. 2025;15:04264.

Figure S1. Location and sequence of chest positions fo auscultation and digital recording of lung sounds

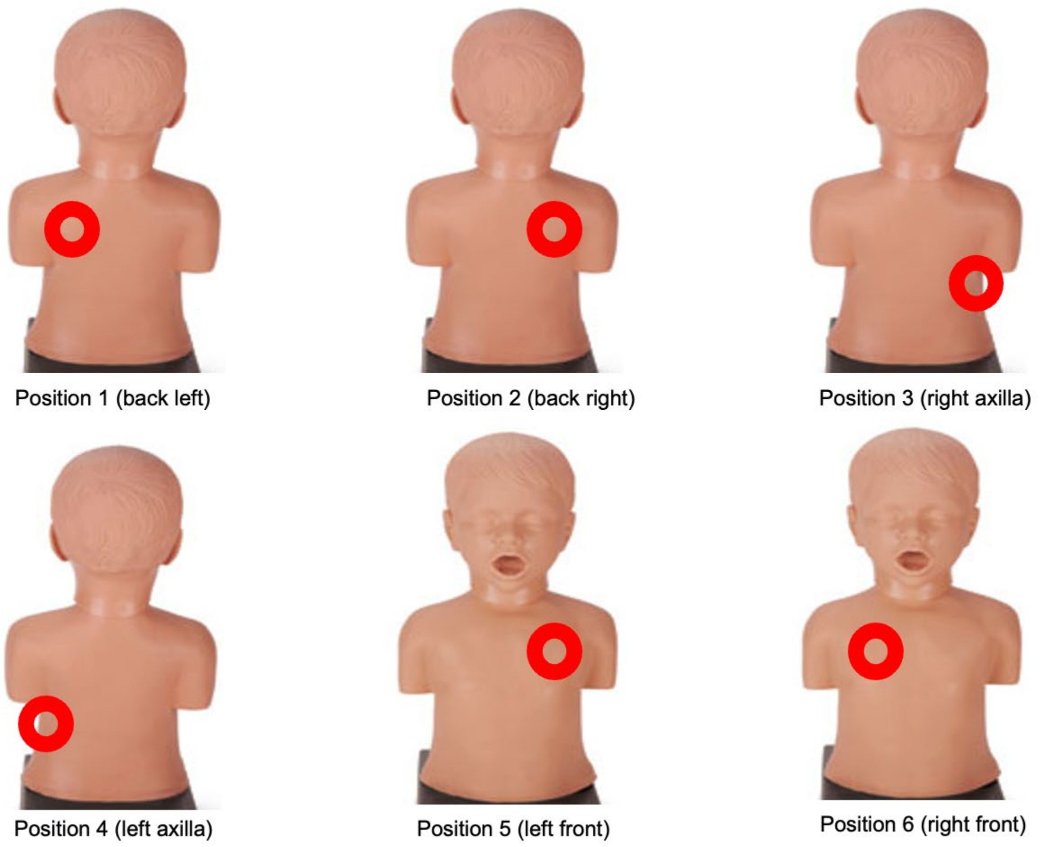

Table S1. Description of demographics and clinical presentation of study population: Participants with interpretable lung sound recordings compared to those with no interpretable lung sound recordings

Characteristic	Interpretable Lung Sound Recordings (N=95)	No Interpretable Lung Sound Recordings (N=5)	P-value
Demographics			
Age in months, median (IQR)	12.6 (5.4–19.0)	13.2 (10.5–15.7)	0.91
Age categories in months, n (%)			
2-11	46 (48%)	2 (40%)	0.93
12-23	32 (34%)	2 (40%)	
24-59	17 (18%)	1 (20%)	
Female	51 (54%)	3 (60%)	0.78
Past medical history			
Prematurity* n (%)	7 (7%)	0 (0%)	0.80
Tuberculosis contacts	6 (6%)	0 (0%)	0.80
History of tuberculosis	1 (1%)	0 (0%)	0.82
Vaccinations up to date†, n (%)	86 (90%)	3 (60%)	0.088
Hospitalization characteristics			
HIV Status			0.62
Infected	0 (0%)	0 (0%)	
Uninfected	77 (81%)	4 (80%)	
Exposed	7 (7%)	0 (0%)	

Unknown	11 (12%)	1 (20%)	
Malaria positive[‡]	11 (12%)[§]	1 (20%)	0.82
Corticosteroid treatment, n (%)	8 (8%)	1 (20%)	0.38
Bronchodilator treatment	21 (22%)	1 (20%)	0.38
Clinical features			
WHO Weight-for-height Z score, mean (SD)	0.3 (1.2)	0.0 (1.6)	0.62
Axillary temperature in Celcius, median (IQR)	36.8 (36.4–37.5)	37.1 (36.9–37.1)	0.99
Respiratory rate in breaths/minute, mean (SD)	54.4 (14.7)	54.0 (13.1)	0.95
SpO_2 in room air, median (IQR)	95.0 (92.0–97.0)	97.0 (95.0–98.0)	0.10

IQR indicates interquartile range; WHO, World Health Organization; SD, standard deviation; SpO_2, peripheral arterial oxyhemoglobin saturation.
*By mother's verbal report.
†Up-to-date if documented in the child's health passport or verbally confirmed by guardian.
‡Positive rapid diagnostic test.
§27 (28%) with missing data.

Table S2. Patient lung sound classification agreement between the physician listening panel and the study clinician and the artificial intelligence algorithm and the study clinician

		Patient Classification N=95
**Physician listening panel and study clinician**		
	Agreement, n (%)	74 (77.9)
	Kappa Statistic (95% CI)	0.332 (0.108–0.556)
	Adjusted Kappa Statistic* (95% CI)	0.558 (0.388–0.728)
**Artificial intelligence algorithm and study clinician**		
	Agreement, n (%)	72 (75.8)
	Kappa Statistic (95% CI)	0.244 (0.022–0.467)
	Adjusted Kappa Statistic* (95% CI)	0.516 (0.340–0.691)

CI indicates confidence interval
*Brennan and Prediger statistic

Table S3. Factors associated with chest position lung sound classification agreement between the physician listening panel and the artificial intelligence algorithm.

Characteristic	Agreement n/N (%)	Disagreement n/N (%)	OR (95% CI)	p Value	Adjusted OR[a] (95% CI)	p Value	Adjusted OR[b] (95% CI)	p Value

All	413/497	84/497						
Age in months (continuous)			0.99 (0.97–1.01)	0.444			0.99 (0.97–1.01)	0.459
Age in months (categorical)								
2-11	198 (47.9)	35 (41.7)	1.0		1.0			
12-23	145 (35.1)	32 (37.1)	0.80 (0.45–1.40)	0.433	0.68 (0.38, 1.21)	0.190		
24-59	70 (17.0)	17 (20.2)	0.73 (0.37–1.46)	0.379	0.78 (0.36, 1.68)	0.522		
Respiratory rate			0.99 (0.98–1.01)	0.480	0.99 (0.98, 1.02)	0.794	0.99 (0.98–1.01)	0.692
Child Uncooperative*								
No	123 (29.8)	15 (17.9)	1.0		1.0		1.0	
Yes	290 (70.2)	69 (82.1)	0.51 (0.27–0.97)	0.040	0.51 (0.25, 1.03)	0.062	0.56 (0.29–1.09)	0.087
Received bronchodilators (n)								
No	316 (76.5)	61 (72.6)	1.0		1.0		1.0	
Yes	97 (23.5)	23 (27.4)	0.81 (0.46–1.43)	0.471	0.86 (0.46, 1.60)	0.629	0.86 (0.46–1.62)	0.642
Received corticosteroids (n)								
No	373 (90.3)	77 (91.7)	1.0		1.0		1.0	
Yes	40 (9.7)	7 (8.3)	1.18 (0.47–2.92)	0.727	1.23 (0.47, 3.22)	0.629	1.22 (0.47–3.18)	0.680

CI indicates confidence interval; OR, odds ratio

Adjusted OR[a] – age modelled as categorical variable
Adjusted OR[b] – age modelled as continuous variable
*Uncooperative was defined as not cooperative throughout the lung sound recording or initially cooperative but progressively not cooperative during the lung sound recording.

Table S4. Factors associated with patient lung sound classification agreement between the physician listening panel and the artificial intelligence algorithm.

**Characteristic**	**Agreement n/N (%)**	**Disagreement n/N (%)**	**OR (95% CI)**	**p Value**	**Adjusted OR[a] (95% CI)**	**p Value**	**Adjusted OR[b] (95% CI)**	**p Value**
All	87/95	8/95						
Age in months (continuous)			0.97 (0.92–1.02)	0.296	-		-	
Age in months (categorical)								
2-11	44 (50.6)	2 (25.0)	1.0		1.0			
12-23	29 (33.3)	3 (37.0)	0.44 (0.07–2.79)	0.384	0.17 (0.02–1.95)	0.156	-	
24-59	14 (16.1)	3 (37.5)	0.21 (0.03–1.40)	0.107	0.03 (0.001–0.50)	0.016	-	
RR			0.97 (0.92–1.02)	0.172	0.94 (0.88–1.00)	0.051	0.95 (0.89–1.00)	0.057
Uninterpretable chest positions								
3 or more uninterpretable chest positions (n)	1 (12.5)	7 (8.1)	0.42 (0.04–4.62)	0.478	-			

2 uninterpretable chest positions (n)	1 (12.5)	7 (8.1)	0.42 (0.04–4.62)	0.478	0.39 (0.03–6.09)	0.504	0.51 (0.03–9.41)	0.649
1 uninterpretable chest positions (n)	3 (37.5)	23 (26.4)	0.46 (0.09–2.46)	0.363	0.15 (0.02–1.52)	0.109	0.23 (0.03–1.79)	0.156
All interpretable chest positions (n)	3 (37.5)	50 (57.4)	1.0		1.0			
Child Cooperative*								
No	25 (28.7)	1 (12.5)	1.0		1.0		1.0	
Yes	62 (81.3)	7 (87.5)	0.35 (0.04–3.03)	0.343	0.92 (0.08–11.20)	0.951	0.83 (0.08–8.80)	0.876
Received bronchodilators prior to or during admission (n)								
No	67 (77.0)	7 (87.5)	1.0		1.0		1.0	
Yes	20 (23.0)	1 (12.5)	2.09 (0.24–18.01)	0.502	5.14 (0.45–59.24)	0.189	4.12 (0.37–46.24)	0.251
Received steroids prior to or during admission (n)								
No	80 (92.0)	7 (87.5)	1.0		1.0		1.0	
Yes	7 (8.0	1 (12.5)	0.61 (0.07–5.71)	0.667	0.51 (0.04–6.78)	0.610	0.29 (0.02–3.78)	0.341

Please note that we have a small number of disagreements to allow us to do meaningful comparison.
CI indicates confidence interval; OR, odds ratio
Adjusted OR[a] – age modelled as categorical variable
Adjusted OR[b] – age modelled as continuous variable
*Child cooperativity was defined as cooperative and quiet or cooperative but vocalizing during auscultation

Table S5. Performance of the artificial intelligence algorithm for detecting abnormal lung sounds in children with World Health Organization-defined severe pneumonia in Malawi using the study physician as reference.

	**Sensitivity, % (95% CI)**	**Specificity, % (95% CI)**	**PPV, % (95% CI)**	**NPV, % (95% CI)**	**LR+ (95% CI)**	**LR- (95% CI)**	**Diagnostic OR (95% CI)**
Chest position classification by study physician	80.3 (75.3–84.7)	87.2 (81.8–91.5)	90.1 (85.8–93.4)	75.3 (69.3–80.7)	6.27 (4.36–9.01)	0.23 (0.18–0.29)	27.7 (16.8–45.7)
Patient classification by study physician	96.3 (89.4–99.2)	66.7 (38.4–88.2)	93.9 (86.3–98.0)	76.9 (46.2–95.0)	2.89 (1.41–5.91)	0.06 (0.02–0.18)	51.3 (11.2–234.0)

CI indicates confidence interval; PPV, positive predictive value; NPV, negative predictive value; LR+, positive likelihood ratio; LR-, negative likelihood ratio; Diagnostic OR, diagnostic odds ratio.
